# Artificial intelligence can be used in the identification and classification of shoulder osteoarthritis and avascular necrosis on plain radiographs: a training study of 7,139 radiograph sets

**DOI:** 10.2340/17453674.2024.40905

**Published:** 2024-06-17

**Authors:** Martin MAGNÉLI, Michael AXENHUS, Johan FAGRELL, Petter LING, Jacob GISLÉN, Yilmaz DEMIR, Erica DOMEIJ-ARVERUD, Kristofer HALLBERG, Björn SALOMONSSON, Max GORDON

**Affiliations:** Department of Clinical Sciences at Danderyd Hospital, Division of Orthopaedics, Karolinska Institutet, Stockholm, Sweden

## Abstract

**Background and purpose:**

Knowledge concerning the use AI models for the classification of glenohumeral osteoarthritis (GHOA) and avascular necrosis (AVN) of the humeral head is lacking. We aimed to analyze how a deep learning (DL) model trained to identify and grade GHOA on plain radiographs performs. Our secondary aim was to train a DL model to identify and grade AVN on plain radiographs.

**Patients and methods:**

A modified ResNet-type network was trained on a dataset of radiographic shoulder examinations from a large tertiary hospital. A total of 7,139 radiographs were included. The dataset included various projections of the shoulder, and the network was trained using stochastic gradient descent. Performance evaluation metrics, area under the receiver operating characteristic curve (AUC), sensitivity, and specificity were used to assess the network’s performance for each outcome.

**Results:**

The network demonstrated AUC values ranging from 0.73 to 0.93 for GHOA classification and > 0.90 for all AVN classification classes. The network exhibited lower AUC for mild cases compared with definitive cases of GHOA. When none and mild grades were combined, the AUC increased, suggesting difficulties in distinguishing between these 2 grades.

**Conclusion:**

We found that a DL model can be trained to identify and grade GHOA on plain radiographs. Furthermore, we show that a DL model can identify and grade AVN on plain radiographs. The network performed well, particularly for definitive cases of GHOA and any level of AVN. However, challenges remain in distinguishing between none and mild GHOA grades.

Plain radiography is the primary imaging modality used in the evaluation of patients with shoulder pain [1]. As the number of radiographic examinations increases worldwide, radiologists face increased workloads, which may impact their diagnostic performance [2]. Early and accurate diagnosis of GHOA allows clinicians to potentially guide patients toward interventions that may manage symptoms of GHOA [3,4].

Another shoulder pathology that has similar symptomatology to GHOA is avascular necrosis (AVN) of the humeral head. Although AVN is a rare condition [5], it remains an important cause of shoulder pain. Similar to GHOA, patients suffering from AVN may benefit from early and accurate diagnosis that helps patient understand their disease and better manage symptoms [6]. Advances in deep learning (DL), a subfield of artificial intelligence (AI), have shown promising results in image interpretation and use in clinical practice [7,8]. This approach has shown significant potential for radiographic image assessment in medicine [2,9-12] and could potentially be used to identify and classify GHOA and AVN on radiographs. We aimed to train a DL model to be used to accurately identify and grade GHOA and AVN on plain radiographs.

We aimed to analyze how a DL model trained to identify and grade GHOA on plain radiographs performs. Our secondary aim was to train a DL model to identify and grade AVN on plain radiographs.

## Methods

### Setting

In this retrospective study, a total of 7,183 plain radiographic examinations in a putative adult population aged 15 years or older were obtained from the Picture Archiving and Communication System (PACS) (GE Healthcare, Chicago, IL, USA) at Danderyd University Hospital. This study adhered to the CLAIM guidelines [13]. All examinations were performed between 2002 and 2016 for clinical purposes, and each examination contained 2–7 radiographs. The variety of radiographs included healthy shoulders, fractures (fractures of the clavicle, humerus, and scapula), shoulders with GHOA, cuff tear arthropathy, or AVN, but did not exclude any diagnosis or radiological findings. The inclusion criteria were all plain radiographic examinations with any combination of “shoulder, scapula, or clavicle.” Shoulder examinations followed a standard plain radiographic protocol with anteroposterior, lateral, and axial projections. Clavicle examinations consisted of 2 frontal projections with a difference of at least 20°. When available, radiologists’ reports were included for each examination, and available for all reviewers. Exclusion criteria were: insufficient quality, missing necessary projections for correct classification, examinations including open epiphyses, and examinations containing data that could in any way compromise anonymity.

### Dataset

The distribution of desired pathologies was aimed to be proportional across the training and validation sets. Directed selection was introduced to obtain an adequate number of examinations representing different grades of GHOA and AVN in each set, based on keywords found in the radiologist’s report. The same patient could appear multiple times when examinations were performed 90 days apart, but there was no overlap between the training, validation, and testing sets. The radiographs used were anonymized and did not contain any patient data.

Examinations in the validation set were initially randomly selected from the original dataset. After periodic assessment of network performance, the radiology reports were searched specifically for examinations with a high probability of the desired pathology and the categories with an insufficient number of cases, or those that had inferior results were added to the validation and training sets. This selection bias was intentionally introduced to ensure a sufficient number of examinations in each category to assess the ability of our network to distinguish between grades within the classification systems:

- Insufficient quality.
- Missing necessary projections for correct classification.
- Examinations including open physes.
- Examinations containing data that could in any way compromise anonymity.

### Diagnosis and classification

Plain radiography is the preferred method for the diagnosis of GHOA and the first modality when assessing a patient with shoulder pain [1,14]. MRI and CT are usually not required for the diagnosis of GHOA, except for the exclusion of other shoulder pathologies or for preoperative investigations [14].

Radiographs can be used to evaluate the joint for narrowing of the joint space, changes in bone anatomy, erosion of the glenoid fossa, subchondral sclerosis of the humeral head and glenoid fossa, subchondral cysts, and osteophytes [14,15].

**Figure 1:**
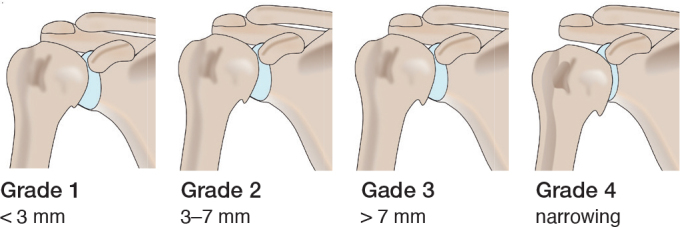
Schematic overview of the Samilson–Prieto classification system.

The Samilson–Prieto (SP) classification system, introduced in 1983 [16], is one of the most used classification systems for GHOA. The SP classification system is simple and demonstrates good inter- and intraobserver reliability [17,18]. In addition, Allain et al. presented a modification of the SP classification system in 1998, in which the severity of GHOA was classified into 4 grades, assessing inferior humeral or glenoid osteophytes or both, narrowing of the GH joint, and sclerosis (Table 1) [19]. Allain’s modified Samilson–Prieto (SPA) classification system also has good interobserver and intraobserver reliability and is considered suitable for scientific purposes (Figure 1) [18].

**Table 1:** SPA classification system according to Allain for GHOA in plain radiographic images with grading definitions

Grade 0	None–normal GH joint.
Grade 1	Mild–inferior humeral or glenoid osteophytes, or both, < 3 mm in height.
Grade 2	Moderate–inferior humeral or glenoid osteophytes, or both, between 3 and 7 mm in height with slight glenohumeral joint irregularity.
Grade 3	Severe–inferior humeral or glenoid osteophytes, or both > 7 mm.
Grade 4	Definitive–narrowing of the glenohumeral joint and sclerosis

In the early stages, AVN shows only subtle changes on plain radiographs. Therefore, the sensitivity of plain radiographs is initially low, whereas MRI shows high sensitivity throughout the disease course [15]. The Cruess [20] classification system for grading AVN in plain radiographic images was introduced in 1976 and is still widely used [5]. The classification consists of grades that evaluate AVN components such as: mottled sclerosis, crescent sign indicating subchondral fracture, progression of subchondral bone collapse, collapse of the terminal humeral head, and involvement of the glenoid (Table 2) [21].

**Table 2:** Cruess classification system for AVN in plain radiographic images with grading definitions

Grade 1	Normal plain radiograph. MRI changes present.
Grade 2	Mottled sclerotic changes present at the humeral head.
Grade 3	Subchondral fracture with mild loss of congruity.
Grade 4	Flattening and collapse of subchondral bone.
Grade 5	Onset of degenerative changes in the glenoid.

### Outcome variables

Outcome variables were classification according to the SPA classification system and the Cruess classification system.

### Review and labeling process

The training and validation datasets were labeled by 3rd and 4th-year medical students from Karolinska Institutet, Stockholm, Sweden. The students were supervised and trained in the review process by 4 senior consultant orthopedic surgeons specializing in shoulder surgery. Several consensus sessions were included in the training. The test set was labeled by the 4 senior consultants. The labels served as a ground truth. The examinations were labeled on a custom-built online platform, where tools to label plain radiographic images based on the SPA and the Cruess classification systems were available. As network training was initiated, the network gradually learned to predict labels for each examination during subsequent labeling. The network’s predictions were incorporated into the online labeling platform and were presented to the human observers as a degree of network certainty ranging between < 50% certainty, 50–70% certainty, or > 70% certainty of selected labels. The human observers had the choice of keeping the network-predicted labels for the particular study or changing the labels based on their own assessment of the study.

The following conditions excluded SPA and Cruess classification:

- Examinations including fractures of the proximal humerus or other deformities to the GH joint.
- Examinations with projections that did not properly enable classification.
- Examinations with any form of GH implant.

As AVN grade I, by definition, is indistinguishable on plain radiographs according to the Cruess classification system, no such label was available. Examinations that were difficult to assign were given a “Revisit” label and later evaluated by a senior orthopedic surgeon. The validation set, containing 561 examinations, was double audited during labeling to ensure consensus of the labels.

### Model architecture and model training

The network used was a modified ResNet type [22], consisting of a total of 35 layers with batch normalization for each convolution layer and adaptive max pool. Stochastic gradient descent was employed to train the network. The labeled images were scaled down, resulting in 256 x 256 pixels to fit the structure of the network, with no zoom applied. Each image was subjected to 80 iterations during training. To increase the robustness of the training, each image was also rotated, flipped, and randomly cropped. A threshold was set for training, and results with fewer than 10 cases in the training set were not trained and consequently were not considered for evaluation.

### Statistics

Statistical analysis was performed using a publicly available version of R (R Foundation for Statistical Computing, Vienna, Austria). Receiver operating characteristics (ROC) curve, area under the ROC curve (AUC) with 95% confidence intervals (CI), sensitivity, specificity, and Youden index (J) were calculated to evaluate the performance of our network for each outcome. A ROC curve is obtained by plotting sensitivity against 1 minus specificity and serves as a probability curve at varied thresholds [23]. AUC shows the ability of the model to discriminate between studies with disease and those without disease [23] and served as the primary outcome measure. AUC is a value between 0 and 1. For this study, we defined AUC between 0.7 and 0.8 as “acceptable,” AUC between 0.8 and 0.9 as “excellent,” and AUC 0.9 or higher as “outstanding,” based on an article by Mandrekar on diagnostic test assessment. An AUC of 1 represents perfect diagnostic accuracy, whereas an AUC of 0.5 indicates a random classifier [23]. AUC was presented as an absolute value with a 95% confidence interval (CI). J summarizes the maximum potential of a diagnostic test and is defined as sensitivity plus specificity minus 1 [24]. J is a value between 0 and 1.

### Ethics, data sharing, funding, and disclosures

An ethical permit has been granted by the Ethical Review Board in Stockholm, Sweden (Dnr 2014/453-31/3). The raw datasets are available from the corresponding author on reasonable request. No funding was received for this study. The authors declare that they have no competing interests. Complete disclosure of interest forms according to ICMJE are available on the article page, doi: 10.2340/17453674.2024.40905

## Results

**Figure 2:**
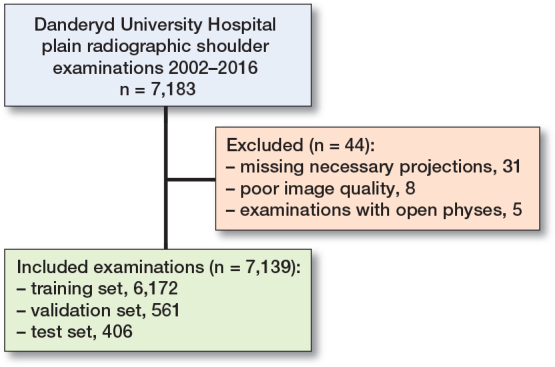
Dataset flowchart with training, validation, and test set.

The dataset after exclusions was divided into 3 subsets for training (n = 6,172), validation (n = 561), and testing (n = 406) (Figure 2).

### Glenohumeral osteoarthritis

**Figure 3:**
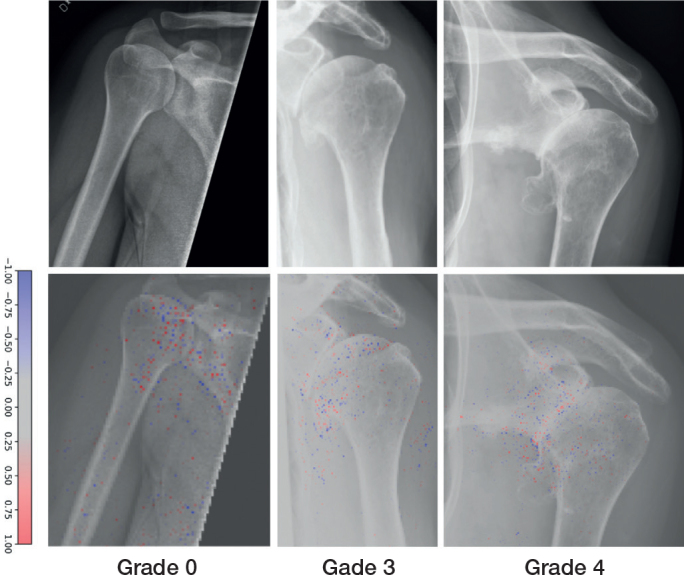
Examples of Samilson–Prieto GHOA grades correctly graded by the network (upper row). Network gradient is provided (lower row).

5,672 (84%) of 6,733 examinations were labelled with an SPA grade (Figure 3). The most common SPA grade was none (n = 4,426), followed by mild (n = 642) (Table 3). The GHOA model achieved acceptable results for all classes with AUC for SPA grades ranging from 0.73 to 0.93 (Table 4).

**Table 3:** Distribution of SPA GHOA grades. Values are count (%)

Grade	Training (n = 6,221)	Validation (n = 562)	Test (n = 308)
None	4,096 (66)	330 (59)	190 (62)
Mild	599 (9.6)	43 (7.7)	35 (11)
Moderate	238 (3.8)	17 (3.0)	10 (3.2)
Severe	162 (2.6)	21 (3.7)	14 (4.5)
Definitive	143 (2.3)	23 (4.1)	59 (19)
Not graded	983 (16)	128 (23)	0 (0)

**Table 4:** Results for all classes with AUC for SPA grades ranging from 0.73 to 0.93

	Cases (n = 308)	Sensitivity (%)	Specificity (%)	Youden’s J	AUC (CI)
None	190	69	85	0.54	0.82 (0.78–0.87)
Mild	35	74	68	0.43	0.73 (0.65–0.80)
Moderate	10	90	79	0.69	0.87 (0.79–0.95)
Severe	14	100	73	0.73	0.85 (0.79–0.91)
Definitive	59	93	86	0.79	0.93 (0.91–0.96)
None or mild	225	72	81	0.53	0.82 (0.78–0.86)
Mild or moderate	45	84	55	0.40	0.76 (0.69–0.82)

### Avascular necrosis

**Figure 4:**
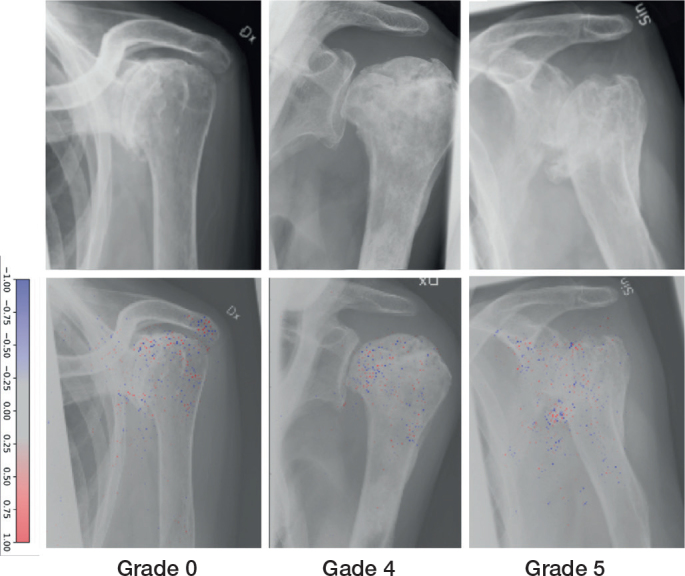
Examples of Cruess avascular necrosis grades correctly labeled by the network (upper row). Network gradient is provided (lower row).

There were a total of 84 examinations of AVN in the combined data, 54 cases in the training set, 8 in the validation set, and 22 in the test set (Figure 4). Grades IV and V were overrepresented in the training set with 27 and 16 cases, respectively, followed by grades II and III. 2 cases were labelled for each grade within the validation set (Table 5). The model achieved outstanding results for all AVN grades with an AUC ranging from 0.90 to 0.94 (Table 6).

**Table 5:** Distribution of AVN grades. Values are count (%)

Grade	Training (n = 6,172)	Validation (n = 561)	Test (n = 406)
2	6 (0.1)	2 (0.4)	2 (0.5)
3	5 (0.1)	2 (0.4)	4 (1.0)
4	27 (0.5)	2 (0.4)	6 (1.5)
5	16 (0.3)	2 (0.4)	8 (2.0)
Total	54 (0.9)	8 (1.4)	22 (5.4)

**Table 6:** All analyzed cases with AVN grades and AUC

Grade	Cases (n = 406)	Sensitivity (%)	Specificity (%)	Youden’s J	AUC (CI)
2	2	100	91	0.91	0.92 (0.89–0.95)
4	6	100	78	0.79	0.90 (0.84–0.97)
5	8	100	80	0.80	0.94 (0.89–0.99)
Total	20	85	93	0.78	0.94 (0.89–0.98)

## Discussion

We aimed to explore the potential of deep learning networks in identifying and grading OA and AVN using plain radiographic images. We found that the network performed well, particularly for definitive cases. However, challenges remain in distinguishing between none and mild GHOA grades.

This is the first study utilizing a DL network to identify and grade GHOA and AVN of the humeral head in plain radiographs. Grauhan et al. have previously shown that AI can be used to detect GHOA although no study has looked into using AI to grade GHOA [25]. Our results suggest that a DL network is a viable approach for this purpose, which is consistent with related studies [2,9-12] that have investigated the identification of OA, OA features, and AVN in other joints and bones. Norman et al. and Kim et al. developed separate DL networks to identify and grade knee OA according to the Kellgren–Lawrence (KL) classification system, demonstrating impressive accuracy [2,9].

Although direct comparison between results based on the KL and SPA classification systems may be difficult because of the lack of strictly defined grades in the KL system [10,12], trends similar to ours are observed in these studies. Consistent with our results, both Norman et al. and Kim et al. showed more accurate network predictions for the highest grade within their respective classification systems, indicating the strength of the DL network in accurately identifying clear cases of OA with pronounced joint space narrowing when anatomy deviates from the norm [2,9]. Similar to our results, both Norman et al. and Chee et al. tended to have poorer results with decreasing OA severity, with the notable exception that both studies achieved high accuracy in identifying the KL equivalent of a healthy joint compared with ours.

### Strengths

We used images acquired according to a standard protocol for plain radiographs. Rather than relying on a single consecutive projection, we evaluated multiple shoulder projections that reflect the clinical reality and with varying image quality, which allowed the network to examine different aspects of the shoulder. This approach may lead to higher generalizability and facilitate more reliable clinical translation.

### Limitations

Medical students performed the labeling of the training and validation data. Novice interpretation of radiographs might influence results. The radiologist’s report accompanying the examinations and close collaboration with experienced supervisors may have compensated for the reviewers’ inexperience. Furthermore, selection bias was introduced by including a variety of pathologies to evaluate the network’s full potential, which resulted in a low number of examinations for some classes.

When we combined the “none” and “mild” grades into a single category, the AUC increased compared with the individual grades, indicating that our network faced challenges in distinguishing between these 2 grades. The ability to distinguish between SPA grades none and mild is critical for early GHOA diagnosis and avoidance of time-consuming and unnecessary additional imaging studies. The difference between the 2 grades is the presence of osteophytes less than 3 mm. Suboptimal projections may be one reason why our network has uncertainty in distinguishing between SPA grade none and mild. The examinations consisted of images in 2–7 shoulder projections, many of which were suboptimal for GHOA assessment, making it difficult for the network to assess small osteophytes. In addition, slightly oblique projections could cause bony structures to appear and be misinterpreted as osteophytes, as described by Norman et al. as an example of network misclassification in their study [9]. Images downsized to 256 x 256 pixels may also explain the difficulty in assessing small osteophytes.

As our network also evaluated shoulder pathologies other than GHOA and AVN, we trained it on complete radiographs. Norman et al. [9] employed focus localization over the joint, by zooming in on the joint space to obtain a limited area for network training. We believe that if we were to train a dedicated network specifically for GHOA identification and grading, a concentrated focus on joint localization could likely enhance performance and improve our results, especially for SPA grades “none” and “mild.” Moreover, Chee et al. and Norman et al. included demographic data and additional patient information to boost network accuracy, whereas we relied solely on image data [2,9].

The network’s performance in differentiating SPA grades “mild” to “severe” is heavily reliant on its capacity to accurately identify and measure the size of osteophytes, which serves as the primary distinguishing feature among these grades. Von Schacky et al. developed a deep learning network for grading the severity of hip OA in radiographs [26]. Their model achieved 83% accuracy for femoral osteophytes and 65% accuracy for acetabular osteophytes in a test set. The authors point out that some radiographic features of OA are more difficult to assess for both deep learning networks and human reviewers. This highlights the challenge of identifying SPA mild to severe grades of GHOA, where accurate assessment of osteophyte size is crucial.

AVN is a rare disease and may present only subtle changes on radiographs [15]. Only a few images were labeled as AVN, probably due to its rarity. Despite the limited number of cases in the training and validation sets, our network showed promising performance in identifying and grading AVN, and we consider an AUC of 0.85 for diagnosing AVN to be an encouraging result. AVN IV and V achieved AUCs of 0.92 and 0.84, respectively, with 100% sensitivity, suggesting that our network can generate relatively accurate predictions even with a limited number of cases in the training and validation sets. Another limitation of our study was the lack of external validation due to the study being a single-center study. External validation of the CNN model should therefore be performed before it is used in a clinical setting. Once trained and validated, a DL network holds promise as a diagnostic aid for radiologists in identifying GHOA and/or AVN. Such a network, capable of discerning a broad range of shoulder disorders, could prove valuable in healthcare settings with constrained resources, such as rural areas or during overnight shifts when access to a radiologist may be limited. Such technology offers the potential to enhance diagnostic accuracy and expedite patient care in underserved communities.

### Conclusion

We found that a DL model can be trained to identify and grade GHOA on plain radiographs. Furthermore, we show that a DL model can identify and grade AVN on plain radiographs. The network performed well, particularly for definitive cases GHOA and any level of AVN. However, challenges remain in distinguishing between none and mild GHOA grades.

## Notes

MM: formal analysis, visualization, writing original draft. MA: investigation, writing original draft. JF, JG, PL: investigation. EDA, YD, KH: investigation, validation. BS: methodology, supervision, visualization. MG: conceptualization, data curation, formal analysis, investigation, methodology, software. All authors: writing, reviewing, and editing.

Handling co-editor: Taco Gosens

*Acta* thanks Walter van der Weegen and other anonymous reviewers for help with peer review of this manuscript.
